# Draft genome sequence of the mucoralean fungus *Syncephalastrum contaminatum* strain 142427, isolated from a human sputum specimen

**DOI:** 10.1128/mra.00857-26

**Published:** 2026-10-22

**Authors:** Yu Feng, Hongxia Wen, Xiaopan Guo, Xinyao Liu

**Affiliations:** 1 Laboratory of Pathogen Research, West China Hospital, Sichuan University 12530, Chengdu, China; Rochester Institute of Technology, Rochester, New York, USA

**Keywords:** sputum, *Syncephalastrum contaminatum*

## Abstract

*Syncephalastrum contaminatum* is a recently described mucoralean fungus with limited genome representation. We report the 26.77 Mb draft genome of strain 142427, isolated from the sputum of a female patient with a lung infection in China. This clinical-source genome supports comparative studies of Syncephalastraceae.

## ANNOUNCEMENT

*Syncephalastrum contaminatum* was described in 2020 and is distinguished from related species using multilocus and genome-scale sequence data, as morphology and internal transcribed spacer (ITS) sequence variation can complicate species identification (1, 2). Broader genome sampling of Mucorales, including *Syncephalastrum* species, is valuable for studies of taxonomy, genome evolution, and pathogenicity-associated traits (3). Here, we report the draft genome sequence of *S. contaminatum* strain 142427, isolated in August 2021 from the sputum of a female patient with a lung infection in Chengdu, China. Although its causal role in disease was not established, this clinical-source genome provides a resource for comparative analyses of *Syncephalastrum* and related fungi.

Strain 142427 was recovered from a sputum specimen on potato dextrose agar (Hopebio, Qingdao, China) at 30°C for 5 days. Microscopic examination suggested that the isolate belonged to the order Mucorales but did not permit species-level identification. Fresh mycelium was mechanically disrupted by glass beads, and genomic DNA was extracted using the QIAamp DNA Blood Mini Kit (catalog no. 51104; Qiagen, Hilden, Germany) according to the manufacturer’s instructions. DNA concentration was measured using a Qubit fluorometer, purity was assessed using a NanoDrop spectrophotometer, and integrity was evaluated by 1% agarose gel electrophoresis. A barcoded paired-end library was prepared from 0.5 µg of DNA using the NEBNext Ultra II DNA Library Prep Kit for Illumina (catalog no. E7645; New England Biolabs, Ipswich, MA, USA), pooled, and sequenced on an Illumina NovaSeq 6000 using an S1 Reagent Kit v1.5 with 150-bp paired-end reads, according to the manufacturer’s recommended protocol. Demultiplexing yielded 31,556,256 reads (15,778,128 read pairs; 4.73 Gb), providing approximately 177× coverage. Unless otherwise stated, default parameters were used. Reads were quality filtered with fastp v1.3.6 (4) and assembled using SPAdes v4.2.0 in isolate mode (5). Assembly statistics were calculated with QUAST v5.3.0 (6). Completeness was assessed using BUSCO v6.1.0 with the fungi_odb10 database (7). Species assignment was assessed by average nucleotide identity (ANI) analysis against available representative Mucorales genomes using skani v0.3.2 (8).

The final draft assembly comprised 1,301 contigs totaling 26,766,572 bp, with an *N*50 of 480,313 bp, an *N*90 of 125,454 bp, an *L*50 of 13, a largest contig of 2,258,251 bp, and 47.34% GC content. BUSCO analysis identified 97.9% complete BUSCOs, including 92.7% single-copy and 5.1% duplicated BUSCOs. Whole-genome comparison with the *S. contaminatum* holotype strain UoMD18-1 (GCA_013461545.1) yielded 97.86% ANI, with 95.78% reference and 96.49% query alignment fractions, supporting assignment to *S. contaminatum*. Genome statistics for strain 142427 and UoMD18-1 are summarized in Table 1. Overall, this genome expands available *S. contaminatum* genomic resources and supports future comparative analyses of Syncephalastraceae diversity.

**TABLE 1:** *Syncephalastrum contaminatum* genome information and assembly quality

Genome statistics	142427	UoMD18-1
Assembly level	Contig	Scaffold
Estimated sequencing depth (×)	177	67
No. of assembled sequences	1,301	373
Largest sequence (bp)	2,258,251	1,658,490
Total length (bp)	26,766,572	26,755,803
GC content (%)	47.34	47.47
*N*50 (bp)	480,313	482,649
*N*90 (bp)	125,454	142,427
*L*50	13	16
*L*90	56	53
Complete BUSCOs (%)	97.9	98.2
Complete single-copy BUSCOs (%)	92.7	92.9
Complete duplicated BUSCOs (%)	5.1	5.3
Fragmented BUSCOs (%)	0.3	0.3
Missing BUSCOs (%)	1.8	1.6
Total BUSCO groups searched	758	758

## Data Availability

The draft genome assembly of *Syncephalastrum contaminatum* strain 142427 has been deposited at DDBJ/ENA/GenBank under the accession no. JCAOFY000000000. The raw sequencing reads have been deposited in the Sequence Read Archive under the accession no. SRR39466267.
